# Microvascular invasion has limited clinical values in hepatocellular carcinoma patients at Barcelona Clinic Liver Cancer (BCLC) stages 0 or B

**DOI:** 10.1186/s12885-017-3050-x

**Published:** 2017-01-17

**Authors:** Cheng Huang, Xiao-Dong Zhu, Yuan Ji, Guang-Yu Ding, Guo-Ming Shi, Ying-Hao Shen, Jian Zhou, Jia Fan, Hui-Chuan Sun

**Affiliations:** 1 Liver Cancer Institute and Zhongshan Hospital, Fudan University, Key Laboratory for Carcinogenesis and Cancer Invasion, the Chinese Ministry of Education, 136 Yi Xue Yuan Rd, Shanghai, 200032 China; 2 Department of Pathology, Zhongshan Hospital, Fudan University, 136 Yi Xue Yuan Rd, Shanghai, 200032 China

**Keywords:** Microvascular invasion, Barcelona Clinic Liver Cancer stage, Hepatocellular carcinoma, Prognosis

## Abstract

**Background:**

Microvascular invasion (MVI) is recognized as a prognostic factor associated with poor outcome in hepatocellular carcinoma (HCC) patients after curative resection. It remains unclear, however, whether MVI can provide prognostic information for patients at a specific tumor stage.

**Methods:**

Consecutive HCC patients who underwent curative resection in years of 2007 and 2008 (discovery cohort) were enrolled in this retrospective study. Patients were stratified by the Barcelona Clinic Liver Cancer (BCLC) staging system. The prognostic significance of MVI for overall survival (OS) and recurrence-free survival (RFS) was studied in each subgroup. The clinical significance of MVI was validated in another cohort of patients underwent curative surgery in the year of 2006 (validation cohort).

**Results:**

Of the 1540 patients in the discovery cohort, 389 (25.3%) patients had detectable MVI. Occurrence rates of MVI in the BCLC stage 0, A, and B subgroups were 12.4, 26.2, and 34.4%, respectively. In univariate analysis, MVI was associated with poor OS and RFS (*P* < 0.001 for both) in HCC patients at stage A, with poor OS in patients at stage 0 (*P* = 0.028), and with poor RFS at stage B (*P* = 0.039). In multivariate analysis, MVI was an independent risk factor for OS (HR = 1.431, 95% CI, 1.163–1.761, *P* < 0.001) and RFS (HR = 1.400, 95% CI, 1.150–1.705, *P* = 0.001) in patients at stage A; and an independent risk factor for RFS (*P* = 0.043) in patients at stage B. A similar clinical significance of MVI was found in the validation cohort.

**Conclusions:**

MVI has limited prognostic value for HCC patients at BCLC stages 0 and B. For those at stage A, MVI was associated with patient survival and may help to select patients with high risk of disease recurrence.

**Electronic supplementary material:**

The online version of this article (doi:10.1186/s12885-017-3050-x) contains supplementary material, which is available to authorized users.

## Background

Liver cancer (mostly hepatocellular carcinoma; HCC) is the second leading cause of cancer-related mortality worldwide [1]. Only approximately 20% of patients with early stage HCC are amenable to curative treatments such as liver resection, liver transplantation, and loco-regional therapies. Although surgical treatments have significantly improved the overall survival, long-term survival is still poor due to high rates of tumor recurrence and metastasis after surgery [2].

Microvascular invasion (MVI) is defined as the presence of tumor cells in portal veins, in large capsule vessels, or in a vascular space lined by endothelial cells [3]. MVI is an early means of cancer cell spread via the vasculature [4]. MVI is only visible on microscopy, and it is difficult to be detected before surgical resection [5]. MVI was found to be one of the most important risk factors for intrahepatic recurrence in HCC patients who underwent curative surgery; thus, it may serve as a surrogate marker reflecting tumor biological characteristics [6, 7], and was recognized as an independent predictor of early recurrence and poor overall survival (OS) following liver resection and liver transplantation [8–10]. However, some authors recently proposed that MVI was not a prognostic factor for all HCC patients. In those with small HCC (≤2 cm), although MVI exhibited excellent prognostic significance [11], it had limited clinical value for treatment and prognosis as compared with the Milan criteria [12]. Thus, whether MVI is associated with patient prognosis only at a specific stage still requires further study.

To date, several HCC staging systems have been proposed to stratify patients into subgroups for better treatment decisions and prognostic prediction [2]. Among these, the Barcelona Clinic Liver Cancer (BCLC) classification is recommended by the American Association for the Study of Liver Diseases (AASLD) and the European Association for the Study of the Liver (EASL) [13, 14]. According to the EASL/AASLD guidelines, hepatic resection is only indicated for BCLC stage 0 or A patients but not for stages B or C. Recently, some authors proposed that the intermediate stage of HCC (BCLC stage B) includes a wide range of patient populations in term of tumor burden and patients’ survival [15]. Several studies have shown that a subset of patients in BCLC stage B will benefit from liver resection over transcatheter arterial chemoembolization (TACE), which is the standard of care for patients at stage B according to EASL/AASLD recommendation [16–18]. Therefore, liver resection is still an important option for patients at stage B in many centers [19–22], as well as in the authors’ institute.

For patients who undergo curative liver resection, pathological findings should be integrated for more precise staging and estimation of risk of tumor recurrence as compared with preoperative stage. For example, MVI could provide additional information for the prognosis prediction and help to select patients with high risk of tumor recurrence for adjuvant therapies. In the current study, we aimed to evaluate whether MVI is an independent risk factor for HCC patients stratified by the BCLC staging system in a cohort of consecutive patents in the authors’ institute, and tried to find the basis for integration of MVI into an existing staging system, the BCLC classification.

## Methods

### Patients

Treatment-naïve patients with histologically diagnosed HCC who underwent curative resection in the authors’ institute between Jan 1, 2007 to Dec 31, 2008 (discovery cohort) were included in this study. Patients with at least one follow-up after surgery were eligible for the present study. Those with preoperative radiologically or intraoperatively diagnosed macrovascular invasion (defined as tumor tissue found in the portal vein, bile duct, or hepatic vein) were excluded from this study. In all cases, preoperative liver function was classified as Child-Pugh class A. Tumor stage was determined according to the BCLC staging system [23]. Tumor cell differentiation was evaluated according to the Edmondson-Steiner classification. MVI status was determined according to histological pathology. The diagnosis of MVI, when tumor cells were detected in microvessels upon microscopic observation, was made based on our established criteria described elsewhere [3]. The data of MVI status were retrospectively retrieved from pathological reports. Patients who underwent curative liver resection in the year of 2006 (validation cohort) were used for the validation of the clinical significance of MVI. This study was approved by the Zhongshan Hospital Fudan University Research Ethics Committee. Informed consent obtained from the patients were written.

### Follow-up and postoperative treatments

All patients were observed until March 2016, with a median observation time of 42.5 months. Follow-up procedures were described in our previous study [24]. Diagnosis of tumor recurrence was based on at least two imaging methods. Treatment modalities after recurrence were administered according to a uniform guideline as described elsewhere [24]. OS was defined as the interval between the date of surgery and death. Recurrence-free survival (RFS) was defined as the interval between the date of surgery and the date of the diagnosis of tumor recurrence or the date of disease-specific death.

### Statistical analysis

Statistical analyses were performed with PASW Statistics 18.0 for Windows (IBM Inc.). In the comparison among different subgroups, quantitative variables were compared using Student’s *t*-test and qualitative variables using the chi-square test or Fisher’s exact test. Kaplan-Meier analysis was used to determine the survival rates. Log-rank test was used to compare patient survival between subgroups, and the Cox regression model was used to perform multivariate survival analysis. All statistical tests were two-sided, and *P* < 0.05 was considered statistically significant.

## Results

### Patient characteristics

A total of 2170 patients were included in the discovery cohort (*n* = 1540) and the validation cohort (*n* = 630) (Table 1). Of the patients in the discovery cohort, 84.7% were male and 81.5% had a history of hepatitis B virus infection, as defined by positive serum hepatitis B surface antigen. The mean tumor size was 5.5 ± 3.5 cm, and 87.1% had a solitary tumor. Because patients with extrahepatic metastasis or macrovascular invasion were excluded in this study, all patients were BCLC stage 0, or A, or B. Of these patients, 22.5% (346/1540) received adjuvant TACE when tumor recurrence was not diagnosed. During a median follow-up of 42.5 months, the median RFS was 50.8 months (95% confidence interval [CI], 45.0–56.7 months) and the median OS was not reached.

**Table 1:** Demographic characteristics of the patients

Features	Discovery cohort (*n* = 1540)	Validation Cohort (*n* = 630)
Age, median (range), year	53.0 (10–86)	53.0 (12–92)
Gender, male/female	1305 (84.7%)/235 (15.3%)	530 (84.1%)/100 (15.9%)
α-Fetoprotein (>200/≤200 ng/dL)	593 (38.5%)/947 (61.5%)	259 (41.1%)/371 (58.9%)
Liver cirrhosis, yes/no/unknown	1268 (82.3%)/251 (16.3%)/21 (1.4%)	512 (81.3%)/102 (16.2%)/16 (2.5%)
Hepatitis B history, yes/no/unknown	1255 (81.5%)/254 (16.5%)/31 (2.0%)	527 (83.7%)/94 (14.9%)/9 (1.4%)
Tumor size, mean ± SD, cm	5.3 ± 3.5	5.5 ± 3.7
Tumor number, multiple/solitary	199 (12.9%)/1341 (87.1%)	78 (12.4%)/552 (87.6%)
Types of resection, anatomic/non-anatomic	1222 (79.4%)/318 (20.6%)	475 (75.4%)/155 (24.6%)
Encapsulation, complete/none/unknown	733 (47.6%)/803 (52.1%)/4 (0.3%)	294 (46.7%)/335 (53.2%)/1 (0.2%)
Tumor differentiation, I–II/III–IV/unknown	1098 (71.3%)/421 (27.3%)/21 (1.4%)	441 (70.0%)/171 (27.1%)/18 (2.9%)
Microvascular invasion, yes/no	389 (25.3%)/1151 (74.7%)	205 (32.5%)/425 (67.5%)
BCLC stage, 0/A/B	194 (12.6%)/1192 (77.4%)/154 (10.0%)	87 (13.8%)/484 (76.8%)/59 (9.4%)

### Correlations between MVI and clinical characteristics

The overall incidence of MVI was 25.3%. Compared to those without MVI, patients with MVI had lower serum albumin (*P* = 0.010), and larger tumor size (*P* < 0.001) (Table 2). Patients with elevated serum α-fetoprotein (AFP > 200 ng/dL; 49.1 vs. 34.9%, *P* < 0.001), large tumor size (>5 cm, 50.1% vs. ≤5 cm, 32.1%, *P* < 0.001), tumors without encapsulation (57.1 vs. 44.6%, *P* < 0.001), poor differentiation of tumor cells (40.1 vs. 23.5%, *P* < 0.001), or advanced BCLC tumor stage (*P*_trend_ < 0.001) had a higher incidence of MVI (Table 2).

**Table 2:** Relationships between microvessel invasion and clinicopathological features in the discovery cohort

Variables	Microvessel invasion		
	Yes (*n* = 389)	No (*n* = 1151)	*P*
Age, year	52.1 ± 11.6	53.3 ± 11.7	0.082
Gender, male/female	332 (85.3%)/57 (14.7%)	973 (84.5%)/178 (15.5%)	0.700
Hepatitis B history, yes/no	317 (83.6%)/62 (16.4%)	938 (83.0%)/192 (17.0%)	0.776
ALT, U/L	54.4 ± 84.7	50.7 ± 64.6	0.370
γ-GT, U/L	94.3 ± 84.9	86.9 ± 92.7	0.168
Albumin, g/L	40.9 ± 4.9	41.6 ± 4.6	0.010
Liver cirrhosis, yes/no	312 (81.0%)/73 (19.0%)	956 (84.3%)/178 (15.7%)	0.153
α-Fetoprotein (>200/≤200 ng/dL)	191 (49.1%)/198 (50.9%)	402 (34.9%)/749 (65.1%)	<0.001
Tumor size, cm	6.5 ± 3.8	4.9 ± 3.3	<0.001
Tumor size (>5 cm/≤5 cm)	195 (50.1%)/194 (49.9%)	370 (32.1%)/781 (67.9%)	<0.001
Tumor number, solitary/multiple	332 (85.3%)/57 (14.7%)	1009 (87.7%)/142 (12.3%)	0.239
Tumor encapsulation, complete/no	166 (42.9%)/221 (57.1%)	637 (55.4%)/512 (44.6%)	<0.001
Tumor differentiation, I–II/III–IV	232 (59.9%)/155 (40.1%)	866 (76.5%)/266 (23.5%)	<0.001
BCLC stage, 0/A/B	24 (6.2%)/312 (80.2%)/53 (13.6%)	170 (14.8%)/880 (76.5%)/101 (8.8%)	<0.001*

*, *P*_trend_. *Abbreviations*: *ALT* alanine aminotransferase, *AST* aspartate aminotransferase, *γ-GT* γ-glutamyl transpeptidase

### Prognostic factors

**Fig. 1:**
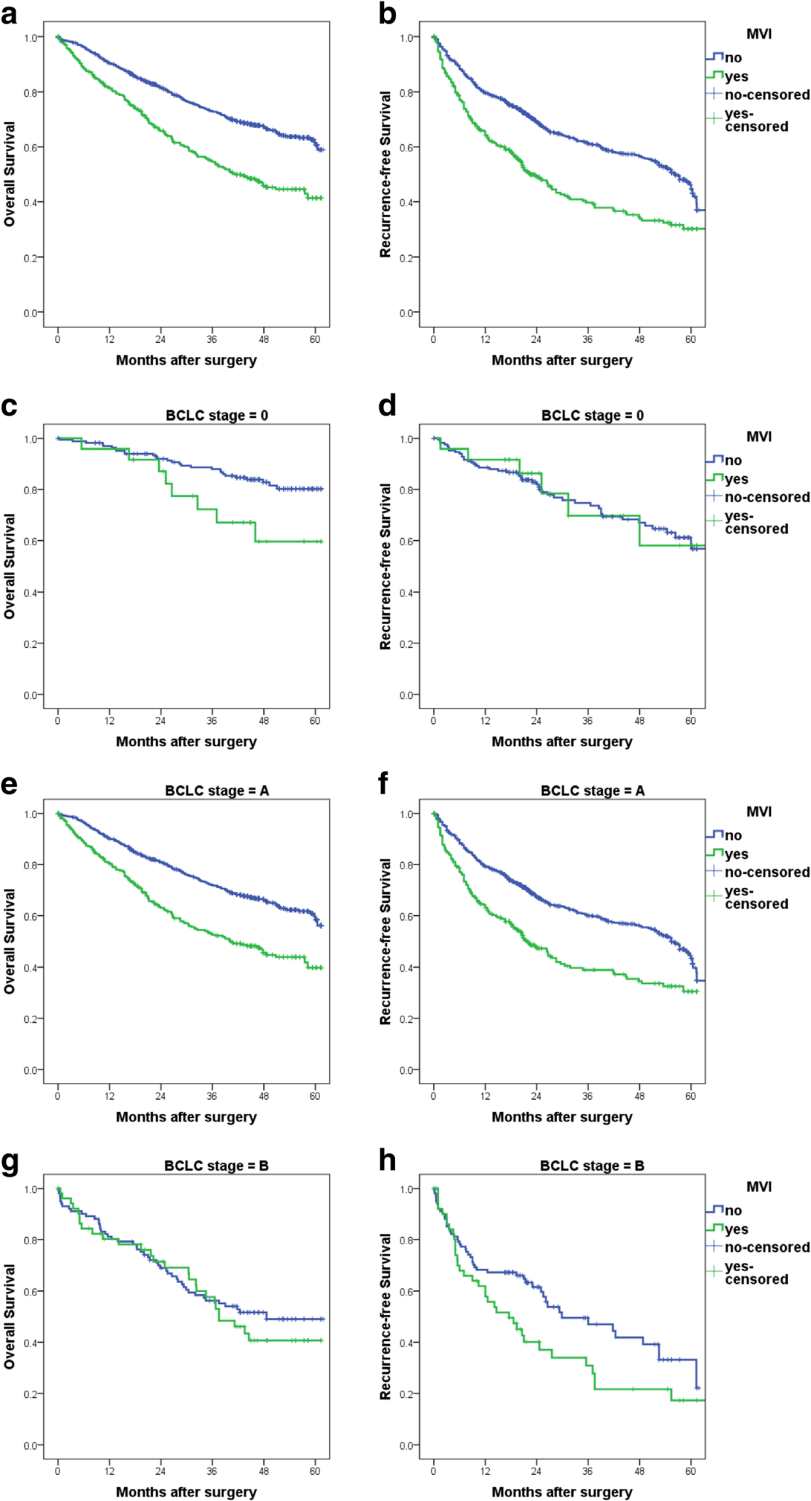
Cumulative overall survival (OS) and recurrence-free survival (RFS) curves of patients with or without microvessel invasion (MVI). MVI was associated with shorter OS and shorter RFS in all the patients without BCLC stratification (**a** and **b**, *P* < 0.001 for both) and in patients at BCLC stage A (**e** and **f**, *P* < 0.001 for both). In patients at BCLC stage 0, MVI was associated with OS but not RFS (**c** and **d**, *P* = 0.028 and *P* = 0.894); and in patients at BCLC stage B, MVI was associated with RFS but not OS (**h** and **g**, *P* = 0.039 and *P* = 0.541)

As shown in Table 3, in univariate analysis, elevated AFP, γ-GT, low serum albumin, large tumor size, multiple tumors, poor tumor cell differentiation, incomplete tumor encapsulation, advanced BCLC stage, and MVI (Fig. 1a, e) were associated with both poor OS and poor RFS. Liver cirrhosis was also associated with poor OS. The features except BCLC stage that showed an association with OS or RFS were adopted for multivariate analysis. MVI was an independent risk factor for both OS (HR = 1.425, 95% CI: 1.187–1.712, *P* < 0.001) and RFS (HR = 1.404, 95% CI: 1.182–1.667, *P* < 0.001) (Table 3).

**Table 3:** Univariate and multivariate analyses of factors associated with survival and recurrence in the discovery cohort

Features	Overall survival				Recurrence-free survival			
	Univariate *P*	Multivariate			Univariate *P*	Multivariate		
		HR	95% CI	*P*		HR	95% CI	*P*
Age, ≤52 vs. >52 year	0.366			NA	0.457			NA
Gender, female vs. male	0.184			NA	0.958			NA
Hepatitis B history, yes vs. no	0.079			NA	0.124			NA
Liver cirrhosis, yes vs. no	0.010	1.493	1.152–1.937	0.002	0.057			NA
α-Fetoprotein, >200 vs. ≤200 ng/dL	<0.001	1.559	1.313–1.851	<0.001	<0.001	1.436	1.227–1.681	<0.001
ALT, >75 vs. ≤75 U/L	0.841			NA	0.313			NA
γ-GT, >50 vs. ≤50 U/L	<0.001	1.420	1.176–1.715	0.002	<0.001	1.331	1.125–1.575	0.001
Albumin, >35 vs. ≤35 g/L	<0.001	0.645	0.501–0.832	0.001	0.003	0.724	0.567–0.926	0.010
Tumor size, >5 vs. ≤5 cm	<0.001	2.052	1.715–2.455	<0.001	<0.001	1.724	1.464–2.029	<0.001
Tumor number, solitary vs. multiple	<0.001	0.683	0.544–0.857	0.001	<0.001	0.666	0.540–0.822	<0.001
Tumor differentiation, III–IV vs. I–II	<0.001	1.275	1.065–1.528	0.008	<0.001	1.290	1.091–1.525	0.003
Tumor encapsulation, complete vs. none	<0.001	1.312	1.107–1.554	0.002	<0.001	1.257	1.076–1.468	0.004
Microvascular invasion, yes vs. no	<0.001	1.425	1.187–1.712	<0.001	<0.001	1.404	1.182–1.667	<0.001
BCLC stage, 0 vs. A vs. B	<0.001			NA	<0.001			NA

*Abbreviations*: *ALT* alanine aminotransferase, *AST* aspartate aminotransferase, *γ-GT* γ-glutamyl transpeptidase, *NA* not adopted, *NS* not significant

### The prognostic value of MVI in subgroups

To study the prognostic value of MVI in patients at a specific tumor stage, we stratified patients with the BCLC staging system. Univariate analysis showed that the presence of MVI was associated with both OS and RFS in patients with early stage HCC (BCLC stage A; Fig. 1). In patients with very early stage (BCLC stage 0), MVI was associated OS but not RFS (Fig. 1c and d, Additional file 1: Table S1; *P* = 0.028 and *P* = 0.894). In patients with intermediate stage HCC (BCLC stage B), MVI was also associated with RFS but not OS (Fig. 1h and g, *P* = 0.039 and *P* = 0.541).

We then evaluated whether MVI remained an independent prognostic factor in BCLC stage A by multivariate analysis (Table 4). MVI remained an independent risk factor for OS (HR = 1.431, 95% CI, 1.163–1.761, *P* < 0.001) and RFS (HR = 1.400, 95% CI, 1.150–1.705, *P* = 0.001) in stage A patients. In stage B patients, MVI was also an independent risk factor for RFS (Additional file 2: Table S2; HR = 1.562, 95% CI, 1.015–2.405, *P* = 0.043).

**Table 4:** Univariate and multivariate analyses of factors associated with survival and recurrence in BCLC stage A patients (*n* = 1192)

Features	Overall survival				Recurrence-free survival			
	Univariate *P*	Multivariate			Univariate *P*	Multivariate		
		HR	95% CI	*P*		HR	95% CI	*P*
Age, ≤52 vs. >52 year	0.751			NA	0.871			NA
Gender, female vs. male	0.434			NA	0.673			NA
Hepatitis B history, yes vs. no	0.025			NS	0.023			NS
Liver cirrhosis, yes vs. no	0.007	1.564	1.181–2.071	0.002	0.030	1.401	1.093–1.796	0.008
α-Fetoprotein, >200 vs. ≤200 ng/dL	<0.001	1.644	1.354–1.996	<0.001	<0.001	1.468	1.227–1.758	<0.001
ALT, >75 vs. ≤75 U/L	0.745			NS	0.494			NA
γ-GT, >50 vs. ≤50 U/L	<0.001	1.327	1.074–1.640	0.009	<0.001	1.246	1.030–1.509	0.024
Albumin, >35 vs. ≤35 g/L	0.001			NS	0.009			NS
Tumor size, >5 vs. ≤5 cm	<0.001	2.080	1.698–2.548	<0.001	<0.001	1.847	1.533–2.225	<0.001
Tumor number, solitary vs. multiple	0.837			NA	0.505			NA
Tumor differentiation, III–IV vs. I–II	<0.001	1.272	1.038–1.559	0.020	<0.001	1.250	1.033–1.514	0.022
Tumor encapsulation, complete vs. none	<0.001	1.279	1.056–1.549	0.012	0.001	1.225	1.026–1.463	0.025
Microvascular invasion, yes vs. no	<0.001	1.431	1.163–1.761	0.001	<0.001	1.400	1.150–1.705	0.001

The prognostic significance of MVI was further evaluated in the validation cohort (Additional file 3: Table S3 and Additional file 4: Table S4). In accordance with the findings in the discovery cohort, MVI was an independent risk factor for both OS and RFS in all the patients (*P* < 0.001 for both). When patients were stratified by BCLC stage, MVI was a risk factor for both OS and RFS for the paints within BCLC A stage (*P* = 0.002 and *P* = 0.003, respectively). In the patients within BCLC B stage, MVI was also an independent risk factor for RFS (*P* < 0.001). In univariate analysis, although MVI showed associations with poor OS in patients within BCLC B stage (Additional file 3: Table S3), and with poor RFS in patients within BCLC 0 stage (Additional file 4: Table S4) it was not an independent risk factor in multivariate analysis.

## Discussion

In the present study we analyzed the presence and prognostic significance of MVI in patients with HCC who underwent curative resection. We found that MVI was an independent risk factor for both OS and RFS. When patients were stratified by BCLC stages, however, MVI was an independent risk factor for OS and RFS in patients at stage A and for RFS in patients at stage B. The results were similar in two independent cohorts.

MVI is a histological feature of HCC related to aggressive behavior of tumor and is widely accepted as one of the most important prognostic factors for patients who undergo curative liver resection or liver transplant. MVI is an early sign of the spread of tumor cells via the peritumoral blood vessels, which was deemed to be a key mechanism of intrahepatic tumor dissemination. In the present study, we found that the presence of MVI in HCC patients increased with tumor progression. Patients with large tumor size, multiple nodules, poor tumor differentiation, or advanced BCLC stages had a higher incidence of MVI (Table 2).

Although MVI is an important risk factor in predicting patient survival after surgery, the present study demonstrated that MVI may only affect the long-term prognosis of patients at specific tumor stages. In HCC patients at BCLC stage 0 (single tumor ≤ 2 cm), MVI was not associated with OS or RFS, a finding that is in accord with previous studies [11, 25, 26]. In patients with very early stage HCC, anatomical resection was more likely to completely remove the tumor-bearing portal territory [27]; therefore, the micro-metastatic nodules infiltrating peritumoral vasculature, namely the MVI, was removed with the tumor nodule. In patients at BCLC stage 0, patients’ age and low albumin were independent risk factors for OS, indicating that none-tumor factors may act as the predominant risk factors that determine the long-term survival in these patients. In patients within BCLC stage A, univariate and multivariate analysis showed that MVI status was associated with both OS and RFS. We think in patients with stage A, MVI as the important tumor biological character, affected the tumor recurrence and overall survival (Table 3). When formulating adjuvant therapeutic strategies, we should take into account patients’ MVI status. In patients within BCLC stage B, MVI was independently associated with RFS but not with OS. For these patients, although MVI increased the risk of tumor recurrence but other factors, e.g., AFP and γ-GT, may undermine its contribution to the long-term survival.

The prevalence of MVI in HCC patients ranged from 15 to 57.1% among 20 different studies [4]. This wide interval is explained not only by geographic variations and the varied features of tumors but also by the lack of consensus on the definition of MVI in HCC. This is also a limitation of this study. The incidence of MVI may have been underestimated in the era before the guideline to detect MVI was established by a cohort of Chinese pathologists [28]. The guideline recommend the detection of MVI on at least 7 points around the tumor nodule from surgery-resected specimens. In order to highlight the association between MVI status and patient prognosis, we chose two cohort of patients in the era (2006–2008) when anti-tumor therapies, e.g., sorafenib, were not widely used in the adjuvant settings. This is a retrospective study in a single center, this is a major limitation of this study, and further investigations are needed in multi-centric studies.

## Conclusions

MVI was an independent risk factor for both OS and RFS in HCC patients who underwent curative liver resection. However, in patients within BCLC stage 0 or stage B, MVI examination could not provide further prognostic information. Only in those at stage A, MVI could determine patient prognosis.

## Additional files

Additional file 1: Table S1. Univariate and multivariate analyses of factors associated with overall survival in BCLC stage 0 patients (*n* = 194) in the discovery cohort. (DOCX 13 kb)
Additional file 2: Table S2. Univariate and multivariate analyses of factors associated with recurrence-free survival in BCLC stage B patients (*n* = 154) in the discovery cohort. (DOCX 13 kb)
Additional file 3: Table S3. Univariate analyses of factors associated with overall survival in the patients from the validation cohort or stratified by BCLC stage. (DOCX 13 kb)
Additional file 4: Table S4. Univariate analyses of factors associated with recurrence-free survival in the patients from the validation cohort or stratified by BCLC stage. (DOCX 13 kb)

## Abbreviations

AASLD: American Association for the Study of Liver Diseases
AFP: α-fetoprotein
BCLC: Barcelona Clinic Liver Cancer
EASL: European Association for the Study of the Liver
HCC: Hepatocellular carcinoma
HR: Hazard ratio
MVI: Microvascular invasion
OS: Overall survival
RFS: Recurrence-free survival
γ-GT: γ-glutamyl transpeptidase
