# Supplementary material for: Microvascular invasion has limited clinical values in hepatocellular carcinoma patients at Barcelona Clinic Liver Cancer (BCLC) stages 0 or B
Source: BMC Cancer. 2017 Jan 17;17:58. doi: 10.1186/s12885-017-3050-x (PMC5240309; doi:10.1186/s12885-017-3050-x)
Supplement: Additional file 1: Table S1. — Univariate and multivariate analyses of factors associated with overall survival in BCLC stage 0 patients (n = 194) in the discovery cohort. (DOCX 13 kb) [file 12885_2017_3050_MOESM1_ESM.docx]

Table S1. Univariate and multivariate analyses of factors associated with overall survival in BCLC stage 0 patients (*n* = 194) in the discovery cohort

| **Features** | **Overall survival** | | | |
| --- | --- | --- | --- | --- |
|  | **Univariate, *P*** | **Multivariate** | | |
|  |  | **HR** | **95% CI** | ***P*** |
| Age, >52 vs. ≤52 y | 0.046 | 1.933 | 1.003–3.725 | 0.049 |
| Gender, female vs. male | 0.265 |  |  | NA |
| Hepatitis B history, yes vs. no | 0.032 |  |  | NS |
| Liver cirrhosis, yes vs. no | 0.350 |  |  | NA |
| α-Fetoprotein, >200 vs. ≤200 ng/dL | 0.023 |  |  | NS |
| ALT, >75 vs. ≤75 U/L | 0.510 |  |  | NS |
| γ-GT, >50 vs. ≤50 U/L | 0.053 |  |  | NA |
| Albumin, >35 vs. ≤35 g/L | 0.001 | 0.331 | 0.149-0.736 | 0.007 |
| Tumor differentiation, III–IV vs. I–II | 0.549 |  |  | NA |
| Tumor encapsulation, complete vs. none | 0.128 |  |  | NA |
| Microvascular invasion, yes vs. no | 0.028 |  |  | NS |
