# Supplementary material for: Microvascular invasion has limited clinical values in hepatocellular carcinoma patients at Barcelona Clinic Liver Cancer (BCLC) stages 0 or B
Source: BMC Cancer. 2017 Jan 17;17:58. doi: 10.1186/s12885-017-3050-x (PMC5240309; doi:10.1186/s12885-017-3050-x)
Supplement: Additional file 2: Table S2. — Univariate and multivariate analyses of factors associated with recurrence-free survival in BCLC stage B patients (n = 154) in the discovery cohort. (DOCX 13 kb) [file 12885_2017_3050_MOESM2_ESM.docx]

Table S2. Univariate and multivariate analyses of factors associated with recurrence-free survival in BCLC stage B patients (*n* = 154) in the discovery cohort

| **Features** | **Recurrence-free survival** | | | |
| --- | --- | --- | --- | --- |
|  | **Univariate, *P*** | **Multivariate** | | |
|  |  | **HR** | **95% CI** | ***P*** |
| Age, >52 vs. ≤52 y | 0.255 |  |  | NA |
| Gender, female vs. male | 0.436 |  |  | NA |
| Hepatitis B history, yes vs. no | 0.608 |  |  | NS |
| Liver cirrhosis, yes vs. no | 0.244 |  |  | NA |
| α-Fetoprotein, >200 vs. ≤200 ng/dL | 0.020 | 1.594 | 1.035-2.455 | 0.034 |
| ALT, >75 vs. ≤75 U/L | 0.865 |  |  | NA |
| γ-GT, >50 vs. ≤50 U/L | 0.253 |  |  | NA |
| Albumin, >35 vs. ≤35 g/L | 0.037 | 0.431 | 0.196-0.946 | 0.036 |
| Tumor size, >5 vs. ≤5 cm | 0.344 |  |  | NA |
| Tumor differentiation, III–IV vs. I–II | 0.496 |  |  | NA |
| Tumor encapsulation, complete vs. none | 0.282 |  |  | NA |
| Microvascular invasion, yes vs. no | 0.039 | 1.562 | 1.015-2.405 | 0.043 |
