# Supplementary material for: Microvascular invasion has limited clinical values in hepatocellular carcinoma patients at Barcelona Clinic Liver Cancer (BCLC) stages 0 or B
Source: BMC Cancer. 2017 Jan 17;17:58. doi: 10.1186/s12885-017-3050-x (PMC5240309; doi:10.1186/s12885-017-3050-x)
Supplement: Additional file 3: Table S3. — Univariate analyses of factors associated with overall survival in the patients from the validation cohort or stratified by BCLC stage. (DOCX 13 kb) [file 12885_2017_3050_MOESM3_ESM.docx]

**Table S3. Univariate analyses of factors associated with overall survival in the patients from the validation cohort or stratified by BCLC stage^*^**

| **Features** | **All patients** | **BCLC 0** | **BCLC A** | **BCLC B** |
| --- | --- | --- | --- | --- |
| Age, ≤52 vs. >52 y | 0.208 | 0.020 | 0.456 | 0.166 |
| Gender, female vs. male | 0.832 | 0.764 | 0.858 | 0.372 |
| Hepatitis B history, yes vs. no | 0.781 | 0.330 | 0.865 | 0.487 |
| Liver cirrhosis, yes vs. no | 0.510 | 0.655 | 0.472 | 0.108 |
| α-Fetoprotein, >200 vs. ≤200 ng/dL | 0.001 | 0.632 | 0.002 | 0.038 |
| ALT, >75 vs. ≤75 U/L | 0.087 | 0.777 | 0.262 | 0.299 |
| γ-GT, >50 vs. ≤50 U/L | <0.001 | 0.011 | <0.001 | 0.032 |
| Albumin, >35 vs. ≤35 g/L | 0.001 | 0.310 | 0.007 | 0.304 |
| Tumor size, >5 vs. ≤5 cm | <0.001 | - | <0.001 | 0.177 |
| Tumor number, solitary vs. multiple | 0.003 | - | 0.278 | - |
| Tumor differentiation, III–IV vs. I–II | <0.001 | 0.235 | <0.001 | 0.612 |
| Tumor encapsulation, complete vs. none | 0.058 | 0.506 | 0.122 | 0.531 |
| Microvascular invasion, yes vs. no | <0.001 | 0.133 | <0.001 | 0.030 |

*, *P* values were shown in this table.
