# Supplementary material for: Microvascular invasion has limited clinical values in hepatocellular carcinoma patients at Barcelona Clinic Liver Cancer (BCLC) stages 0 or B
Source: BMC Cancer. 2017 Jan 17;17:58. doi: 10.1186/s12885-017-3050-x (PMC5240309; doi:10.1186/s12885-017-3050-x)
Supplement: Additional file 4: Table S4. — Univariate analyses of factors associated with recurrence-free survival in the patients from the validation cohort or stratified by BCLC stage. (DOCX 13 kb) [file 12885_2017_3050_MOESM4_ESM.docx]

**Table S4. Univariate analyses of factors associated with recurrence-free survival in the patients from the validation cohort or stratified by BCLC stage^*^**

| **Features** | **All patients** | **BCLC 0** | **BCLC A** | **BCLC B** |
| --- | --- | --- | --- | --- |
| Age, ≤52 vs. >52 y | 0.159 | 0.040 | 0.362 | 0.214 |
| Gender, female vs. male | 0.149 | 0.880 | 0.097 | 0.129 |
| Hepatitis B history, yes vs. no | 0.370 | 0.117 | 0.920 | 0.208 |
| Liver cirrhosis, yes vs. no | 0.249 | 0.600 | 0.191 | 0.002 |
| α-Fetoprotein, >200 vs. ≤200 ng/dL | 0.009 | 0.366 | 0.019 | 0.018 |
| ALT, >75 vs. ≤75 U/L | 0.284 | 0.493 | 0.443 | 0.153 |
| γ-GT, >50 vs. ≤50 U/L | <0.001 | 0.029 | <0.001 | 0.031 |
| Albumin, >35 vs. ≤35 g/L | 0.025 | 0.288 | 0.044 | 0.873 |
| Tumor size, >5 vs. ≤5 cm | <0.001 | - | 0.001 | 0.187 |
| Tumor number, solitary vs. multiple | 0.001 | - | 0.599 | - |
| Tumor differentiation, III–IV vs. I–II | 0.010 | 0.649 | 0.009 | 0.483 |
| Tumor encapsulation, complete vs. none | 0.071 | 0.841 | 0.071 | 0.787 |
| Microvascular invasion, yes vs. no | <0.001 | 0.041 | <0.001 | 0.004 |

*, *P* values were shown in this table.
